# High levels of untreated distress and fatigue in cancer patients

**DOI:** 10.1038/sj.bjc.6601887

**Published:** 2004-05-25

**Authors:** L E Carlson, M Angen, J Cullum, E Goodey, J Koopmans, L Lamont, J H MacRae, M Martin, G Pelletier, J Robinson, J S A Simpson, M Speca, L Tillotson, B D Bultz

**Affiliations:** 1 Department of Psychosocial Resources, Tom Baker Cancer Centre, Alberta Cancer Board, Calgary, Alberta, Canada; 2 Department of Oncology, Faculty of Medicine, University of Calgary, Calgary, Alberta, Canada; 3 Department of Social Work, University of Calgary, Calgary, Alberta, Canada; 4 Department of Psychiatry, University of Calgary, Calgary, Alberta, Canada

**Keywords:** Neoplasms, distress, psychosocial screening, fatigue, counselling

## Abstract

The purpose of the study was to assess a large representative sample of cancer patients on distress levels, common psychosocial problems, and awareness and use of psychosocial support services. A total of 3095 patients were assessed over a 4-week period with the Brief Symptom Inventory-18 (BSI-18), a common problems checklist, and on awareness and use of psychosocial resources. Full data was available on 2776 patients. On average, patients were 60 years old, Caucasian (78.3%), and middle class. Approximately, half were attending for follow-up care. Types of cancer varied, with the largest groups being breast (23.5%), prostate (16.9%), colorectal (7.5%), and lung (5.8%) cancer patients. Overall, 37.8% of all patients met criteria for general distress in the clinical range. A higher proportion of men met case criteria for somatisation, and more women for depression. There were no gender differences in anxiety or overall distress severity. Minority patients were more likely to be distressed, as were those with lower income, cancers other than prostate, and those currently on active treatment. Lung, pancreatic, head and neck, Hodgkin's disease, and brain cancer patients were the most distressed. Almost half of all patients who met distress criteria had not sought professional psychosocial support nor did they intend to in the future. In conclusion, distress is very common in cancer patients across diagnoses and across the disease trajectory. Many patients who report high levels of distress are not taking advantage of available supportive resources. Barriers to such use, and factors predicting distress and use of psychosocial care, require further exploration.

Psychosocial distress in cancer patients has been identified as a significant and ongoing problem. The National Comprehensive Cancer Network (NCCN) Distress Management Panel has defined distress as

…a multi-determined unpleasant emotional experience of a psychological (cognitive, behavioral, emotional), social, and/or spiritual nature that may interfere with the ability to cope effectively with cancer, its physical symptoms and its treatment. Distress extends along a continuum, ranging from common normal feelings of vulnerability, sadness and fears to problems that can become disabling, such as depression, anxiety, panic, social isolation, ad spiritual crisis. (National Comprehensive Cancer Network, 2002)

Previous studies have documented that approximately one-third of all oncology patients will experience significant levels of distress associated with diagnosis and treatment of cancer, which warrants psychosocial treatment (Derogatis *et al*., 1983; Stefanek *et al*., 1987; Zabora *et al*., 1997; Sellick and Crooks, 1999; Zabora *et al*., 2001a; Carlson and Bultz, 2003b). Also well documented, using rigorous methodology, is the ability of various psychosocial treatments to alleviate distress levels and improve quality of life in cancer patients, reviewed in several papers and meta-analyses (e.g. (Cunningham, 1995; Meyer and Mark, 1995; Bottomley, 1997; Fobair, 1997; Iacovino and Reesor, 1997; Fawzy *et al*., 1998; Fawzy, 1999; Blake-Mortimer *et al*., 1999; Cunningham, 2000; Schneiderman *et al*., 2001; Carlson and Bultz, 2003a).

Interventions usually assume one of four common forms: psychoeducation, cognitive-behavioural training (group or individual), group supportive therapy, and individual supportive therapy. As well, they are usually targeted to one of three points on the illness trajectory: diagnosis/pretreatment, immediately post-treatment or during extended treatment (such as radiotherapy or chemotherapy), and disseminated disease or death (Schneiderman *et al*., 2001). Certain modalities of treatment have been shown to be more efficacious at one or more of these time periods. For example, psychoeducation may be most effective during the diagnosis/pretreatment time period, when patient information needs are high. However, for later stage adjustment with more advanced disease, group support may be more effective (Blake-Mortimer *et al*., 1999), while cognitive-behaviour techniques such as relaxation, stress management and cognitive coping may be most useful during extended treatments (Fawzy, 1995; Bottomley, 1997).

Several agencies, both American and International, have developed guidelines for psychosocial care, which include screening guidelines (see Carlson and Bultz (2003b) for an overview of distress screening issues). The Canadian Association of Psychosocial Oncology has published a book of Standards which details principles of practice, professional issues, and organization and structure of psychosocial oncology programmes (Canadian Association of Psychosocial Oncology, 1999). Principle 7 states that ‘psychosocial service needs of patients and families are assessed systematically using appropriate tools’ (p. 5). The National Comprehensive Cancer Network (NCCN) and the American Society of Clinical Oncology (ASCO) also have guidelines regarding the identification and management of distress (available at: http://www.nccn.org/physician_ gls/index.html). The NCCN guidelines were developed by a Distress Management Panel that included many researchers and clinicians directly involved in major American screening programmes. The standards of care developed by this group state that: ‘All patients should be screened for distress at their initial visit, at appropriate intervals, and as clinically indicated.’ (DIS-3) (National Comprehensive Cancer Network, 2002). They go on to delineate clinical practice guidelines for the treatment of distress.

Where there has often been a disconnect, however, is in the ability of psychosocial oncologists to use information about distress levels obtained through routine screening of patients, and to direct those patients identified as in need of services to the appropriate sources of care. This idea of screening followed by appropriate triage is not new in psychosocial oncology, but rarely happens in routine clinical practice. Researchers at Johns Hopkins University have developed a comprehensive psychosocial oncology screening programme that assesses all patients at the point of entry into the cancer care system. Based on this assessment of emotional distress and practical problems, the patients who need support are contacted personally within 48 h, and those who do not indicate significant current need are provided with information regarding the options for social and psychological care for future consultation (Zabora *et al*., 2001b).

Unfortunately, even routine psychosocial screening of patients is not the norm in terms of care of cancer patients. More commonly, patents are either self-referred, or referred by a member of the medical treatment team who becomes alerted to the patient's distress in the course of clinical care. This can potentially result in missing a large proportion of patients who may be in need of care, but who are either too distressed or without the instrumental resources to find their way through the often confusing medical system to reach psychosocial care. In addition, patients themselves may be so preoccupied with the physical components of their cancer that they may be unaware of the seriousness of the level of their own psychosocial distress, and potentially unaware that help is available to treat these symptoms. It may be the case, then, as a result of this unsystematic referral system, that the most disadvantaged patients may be the least likely to get necessary care.

In order to investigate this possibility, and also to comprehensively assess the most common psychosocial problems reported by a population of patients, we sought to assess every patient who visited a large urban tertiary cancer centre over a 4-week period of time in January of 2003. Patients with any diagnosis and all treatment stages were included in the screening programme, in order to capture a cross-sectional snapshot of patients that could be generalised to the cancer centre population at large. A substantial sample would also allow comparisons between men and women, patients from different ethnic backgrounds, with different types of cancer, and at varying stages of treatment.

## METHODS

### Subjects and setting

All patients over the age of 18 years who visited the Tom Baker Cancer Centre for any reason (diagnosis, new patient consultation, treatment, follow-up) during the course of the concentrated screening effort were eligible to participate in the screening programme. No restrictions were placed on age, ethnicity, gender, type of cancer, stage of illness, or disability. Patients who were unable to communicate in English were noted as such and an incomplete questionnaire was submitted with this proviso. It was ensured that each patient was assessed only once.

The Tom Baker Cancer Centre is a large regional tertiary cancer centre that serves a population of approximately 2 million people, with approximately 3500 new patients yearly, and 32 000 annual patient visits. The Department of Psychosocial Resources at the TBCC is one of the largest in Canada, employing six full-time and two part-time psychologists, two full-time and two part-time clinical social workers, as well as a number of research staff.

### Instruments

#### Demographic and Cancer History Questionnaire

The first few questions collected standard demographic and disease-related information.

#### Psychosocial Questionnaire

**Figure 1:**
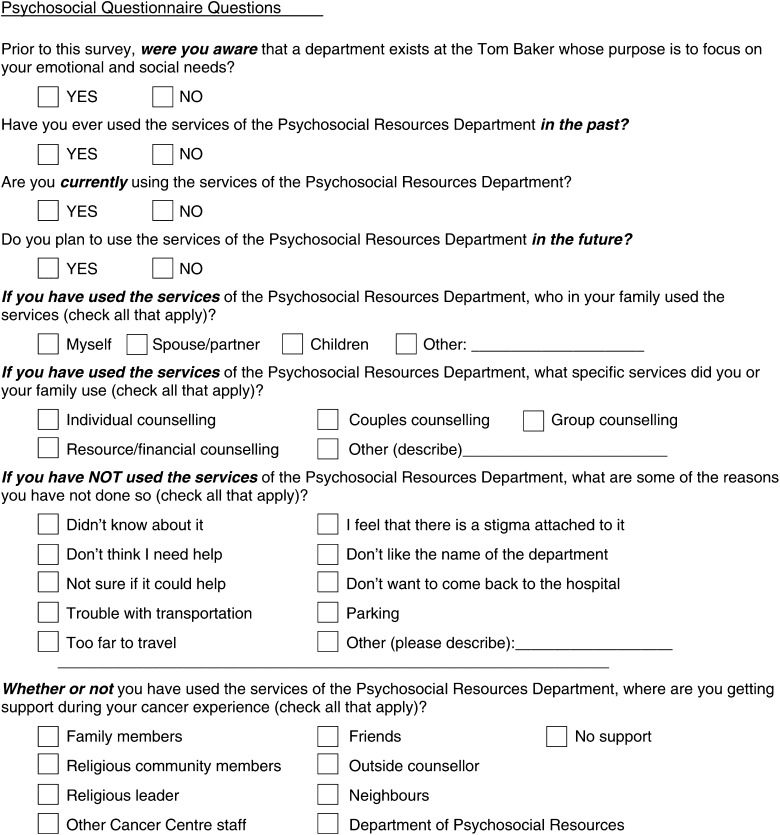
Psychosocial Questionnaire Questions.

The next section asked a series of questions regarding awareness and use of the Department of Psychosocial Resources at the TBCC. Questions were developed by the psychosocial staff and pretested on a group of patients for clarity and ease of understanding. Included questions are listed in Figure 1

.

#### Problem checklist

The following section included the patient problem checklist with 22 items, adapted from that used by the Johns Hopkins research group to fit the local setting. Patients were instructed to check off all problems they currently have or expect to have.

#### Brief Symptom Inventory – 18 (BSI–18)

Published in 2001 (Derogatis, 2001), this short 18-item instrument is the latest in an integrated series of test instruments designed to measure psychological distress. Shortened from the original Symptom Checklist (SCL-90-R; Derogatis, 1983), the 53-item Brief Symptom Inventory (BSI) (Derogatis, 1993), and now the 18-item version have been normed for use with cancer patients. The BSI and the SCL-90 have been used extensively with medical populations, including cancer patients, and have demonstrated high levels of sensitivity and specificity in screening for psychological distress (Zabora *et al*., 1990;Carlson and Bultz, 2003b). The BSI-I8 was developed specifically as a highly sensitive and efficient screen for psychiatric disorders and psychological distress. The BSI-18 is highly reliable ad valid and is significantly correlated with the BSI. The instrument yields three subscale scores: Somatization, Depression and Anxiety, with internal consistencies ranging from 0.74 to 0.89, and correlations with the BSI ranging from 0.91 to 0.96. A composite score, the General Severity Index (GSI), also shows similar high levels of reliability. Average completion time is 1–3 min. The BSI-18 is the version that the Johns Hopkins group has been recently using in their screening programme, and on which they have published norms from a large sample of patients (Zabora *et al*., 2001b).

In addition to continuous scoring on each subscale, cutoff scores are recommended for ‘caseness’ of each subscale and the GSI scores for cancer patients, based on the guidelines in the manual. Individuals who score at or over the cutoff values are considered to be experiencing levels of distress that require psychosocial intervention. Criteria for ‘caseness’ vary between men and women for anxiety and somatization, but not for depression.

### Procedures

The assessments were collected over a 1-month period as a ‘snapshot’, or cross-sectional analysis of all patients visiting the TBCC during this time period, both new and follow-up, across all clinics. Ethical approval was obtained from the local Research Ethics Board, and all affected departmental managers consented in writing to cooperate in the data collection.

Four main departments were targeted with a view to maximising patient accrual: (a) Outpatients; (b) Medical Daycare; (c) Radiation Therapy; and(d) Laboratory. Six staff were stationed in the four departments on a full-time basis during the month of data collection. Patients were approached when they arrived in each area, and given the assessment package to complete in the waiting area. The package consisted of a patient information sheet for them to keep (signed informed consent was not required by the local Research Ethics Board, as all responses were provided in anonymity), the questionnaires, an envelope, and a pin consisting of a ribbon with a ‘happy face’ sticker. Research assistants were available to help patients complete the packages and answer questions.

If patients chose not to complete the questionnaires, they were asked to check off their reason for declining at the top of the questionnaire (or the RA asked about their reason and checked the box for them) and the incomplet questionnaires were submitted. Once a patient had completed and submitted the questionnaires, they were asked to wear the ‘happy face’ pin when at the centre, to alert research assistants not to approach that patient again. Packages were collated and counted at the end of each day and stored in locked filing cabinets for later data analysis.

## RESULTS

### Subjects

Questionnaires were collected from 3095 patients over the course of the 4 weeks of screening. Of these, 319 (approx. 10%) declined to complete the questionnaires. The reasons for this include the following: not interested (43.5% of decliners), cannott read English (13.2%), feeling too sick (11%), too rushed (10.7%), too tired (6.6%), and other (15.4%). This left a sample of 2776 patients (90%) who completed the questionnaires. The statistics kept by the TBCC for visits in the month of January indicated that there were 750 new patient visits, and 3015 follow-up visits, for a total of 3765 patient visits. This would indicate that we captured 82.2% of all patients, with full data on 73.7%. However, these numbers may be conservative estimates, as the statistics report visits, not individual patients. Therefore, if a patient visited more than once during the month, each visit would be recorded as a separate statistic.

Demographic characteristics of the sample are presented in Table 1

**Table 1:** Demographics

	**Mean (years)**	**s.d.**
Age	60.00	14.69
Education	13.18	3.42
Duration of illness	3.26	4.59
		
	**Number**	**Valid percentage**
Gender		
Female	1451	52.2
Ethnicity		
European	896	32.3
Canadian	703	25.4
British	571	20.6
E and SE Asian	121	4.4
Aboriginal	31	1.1
South Asian	27	1.0
Ethnicity missing	349	12.6
Ethnicity other	74	2.7
Income		
<10 000	145	5.2
10 000–20 000	354	12.8
20 000–40 000	659	23.8
40 000–60 000	493	17.8
60 000–80 000	316	11.4
80 000–100 000	203	7.3
<100 000	296	10.7
Missing	306	11.0

. The sample was 52.2% of female subjects, an average age of 60 years, with mean levels of 13 years education, and 3 years postdiagnosis. They were primarily from European, British, or Canadian origins (78.3%), and income was fairly equally distributed, with 41.8% earning less than $40 000 year annual household income, 29.2% between $40 and $80 thousand, and 18% earning over $80 000.

In terms of cancer type, the largest group was breast cancer (23.5%), followed by prostate (16.9%), colorectal (7.5%), and lung (5.8%). Most other types of cancer were also represented, as summarised in Table 2

**Table 2:** Type of cancer

**Type**	**Number**	**Percentage**
Breast	652	23.5
Prostate	469	16.9
Colorectal	209	7.5
Lung	162	5.8
Lymphoma (non-Hodgkin's)	156	5.6
Leukemia	109	3.9
Ovarian	88	3.2
Brain	81	2.9
Multiple meyeloma	70	2.5
Melanoma	67	2.4
Testicular	58	2.1
Cervix	53	1.9
Uterine	44	1.6
Hodgkin's disease	41	1.5
Other	430	15.5
Missing	83	3.0

. The primary reason for the visit was identified as follow-up in 51.9% of the cases, with tests (10.9%), chemotherapy (9.6%), and radiotherapy (9.0%) being the next most common. 7.7 % of patients were at the Centre for their first visit, and the remainder were either picking up medication (1.6%), had another reason (8.7%), or did not check off a reason for the visit (0.7%).

### Distress

The mean scores for the three subscales and the GSI on the BSI-18 are presented in Table 3

**Table 3:** BSI-18 scores overall and by gender

	**Mean score**	**s.d.**	**Percent meeting caseness criteria**
Somatisation	4.03	4.21	39.7
Men	3.77	4.17	42.2^a^
Women	4.26	4.23	37.4
			
Depression	3.59	4.48	36.3
Men	3.25	4.34	32.2^a^
Women	3.88	4.58	39.8
			
Anxiety	3.83	4.53	30.3
Men	3.12	4.08	31.5
Women	4.46	4.80	29.3
			
GSI	11.34	11.52	37.8
Men	10.03	11.01	37.6
Women	12.49	11.84	37.9

a Men statistically different per cent of cases than women, *P*<0.01.

. The mean scores as well as the percentage of each group meeting the criteria for ‘caseness’ are displayed. Overall, close to 38% of the entire sample met the caseness criteria for Global Severity, with some variations in the prevalence of each subscale such that overall somatisation was the most common (39.7%), followed by depression (36.3%), and anxiety (30.3%). Men were more likely than women to be cases of somatisation (*χ*^2^=5.85, *P*<0.05), whereas women were more likely to be cases of depression (*χ*^2^=15.64, *P*<0.001). No gender differences in anxiety or the overall severity were found.

Further investigation of distress levels by ethnicity, income, type of cancer, and reason for visit are presented in Table 4

**Table 4:** BSI GSI scores by demographic and disease variables

	**Mean score**	**s.d.**
Ethnicity		
Canadian/British/European	10.6	10.8
Other	15.4^a^	13.7
		
Income		
<40 000	12.4^b^	12.0
>40 000	9.9	10.4
		
Type of cancer		
Breast	12.0	12.2
Prostate	7.3^c^	8.8
Other	11.8	11.2
		
Reason for visit		
First visit	10.6	9.4
Active treatment	11.8^d^	11.8
Follow-up	10.6	11.1

a Other ethnicities more distressed than Canadian/British/European.

b Lower income more distressed than higher.

c Breast and other diagnoses more distressed than prostate.

d Active treatment more distressed than follow-up patients.

. For these analyses, continuous GSI scores were compared between the categorical groups using ANOVA. Patients representing minority ethnicities were more distressed than those of Canadian/British/European descent (*F*=27.98, *P*<0.001), as were those from lower income households (*F*=24.63, *P*<0.001). Patients with prostate cancer were less distressed than patients with breast cancer (*P*<0.001) and patients with other diagnoses (*P*<0.001; overall *F*=35.32), and those on active treatment were more distressed than those on follow-up treatment (*F*=3.72, *P*<0.05).

The association of these variables with distress scores from a multivariate perspective was also explored using multiple linear regression analyses. The demographic variables of age, gender, education, ethnicity (Canadian/British/European *vs* Other), and income (<40 000 *vs* <40 000) were first added in one block, followed by the disease-related variables of the type of cancer (breast or other), duration of illness, and reason for visit (follow-up or other). All of these variables were included in the final best fitting model (*F*=12.33, *P*<0.001), except years of education and type of cancer. Possibly, the dummy variables in the cancer category of breast or other were too broad to detect differences. Thus, variables in the model included: age (*t*=−5.36, *P*<0.001), sex (*t*=2.91, *P*<0.001), ethnic background (*t*=4.17, *P*<0.001), income (*t*=−5.38, *P*<0.001), duration of illness (*t*=2.40, *P*<0.05), and reason for visit (*t*=−2.60, *P*<0.01). Directionality indicated that younger age, female sex, ethnicity other than Canadian/British/European, lower income, longer duration of illness, and not being on follow-up were all independently associated with higher distress levels. Longer duration of illness, in this case, may possibly be a proxy for more advanced disease or disease recurrence.

### Distress comparisons by disease site

Distress levels in this sample were compared to those reported on the BSI-18 from the Johns Hopkins Oncology Centre as published by Zabora *et al*. (2001a). These are displayed in Table 5

**Table 5:** Percentage of distress ‘cases’ by cancer diagnosis

**Site**	**Johns Hopkins (Zabora *et al*, 2001a)**	**TBCC**
Lung	43.4	57.6
Brain	42.7	45.3
Hodgkin's	37.8	48.7
Pancreas	36.6	52.2
Lymphoma	36.0	42.5
Liver	35.4	NA
Head and Neck	35.1	48.6
Adenocarcinoma (unknown primary)	34.9	NA
Breast	32.8	35.4
Leukaemia	32.7	45.5
Melanoma	32.7	34.4
Colon	31.5	32.1
Prostate	30.5	26.6
Gynaecological	29.6	38.3

. The Pearson product–moment correlation coefficient between Johns Hopkins and Tom Baker values was highly significant in a positive direction (*r*=0.764, *P*<0.001), indicating a high degree of similarity in the distress levels of patients within different cancer diagnoses at both institutions. Lung patients endorsed the highest levels of distress, followed by a cluster containing pancreatic, Hodgkin's lymphoma, brain, head and neck, leukaemia, and lymphoma. The cluster of diagnoses with the lowest levels of distress included gynaecological, breast, melanoma, colon and prostate cancers.

### Common problems

**Figure 2:**
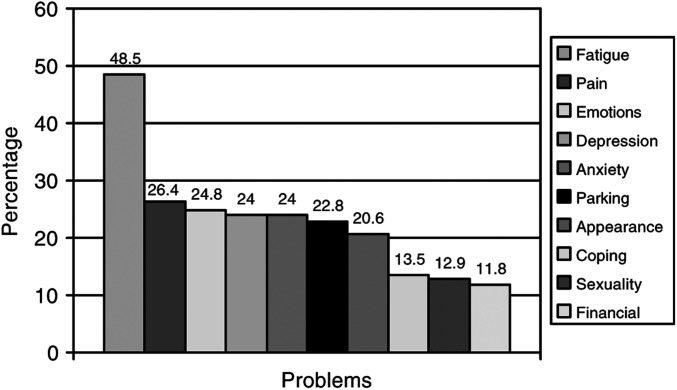
Problems endorsed (percentage of respondents).

The top problems endorsed by patients are depicted in Figure 2

. The most frequent problem endorsed was fatigue (48.5%), followed at much lower rates, almost equally by pain (26.4%), managing emotions/stress (24.8%), depression (24.0%), and anxiety (24.0%). The modal number of problems endorsed by patients was 3, and the correlation between number of problems endorsed and BSI-18 GSI score was *r*=0.57, *P*<0.001.

### Awareness and use of psychosocial resources

When asked if they were aware of the existence of a specific department whose purpose was to address the emotional and social needs of patients, 68.1% indicated awareness of such a department. When looked at by reason for visit, more patients on active treatment (72.1%), and follow-up (73.9%), than those on their first visit (49.8%) were aware of the Psychosocial Resources Department. In terms of usage, 17.8% indicated use of the department in the past, 6.7% endorsed currently using the services, and 20.4% said they planned to use psychosocial resources in the future. These three combined (past, current, and future users) constituted 36.4% of the sample.

In terms of who used the services, most commonly it was the patients themselves (18.8% of total sample), followed by a spouse (7.4%) and children (2.6%). The highest use of services was for individual counselling (14.5%), followed by couples counselling (4.7%), group (3.0%), and resource/financial (3.0%).

Patients who did not use the service were also asked the reasons for not using it. The main reason for not using Psychosocial Resources was the perception of not needing any help (44.1% of those who replied to this question), followed by not knowing about the services provided (19.2%). Of note is that almost no patients reported the name of the department (0.4%), or any stigma attached to it (0.7%), as a deterrent to using the services. Other reasons included it being too far to travel (8.5%), parking problems (8.1%), and not being sure if the services would help (8.4%).

Use of the Psychosocial Department was also analysed by distress caseness, cancer site, ethnicity, and reason for visit. Patients with breast cancer were higher utilisers of psychosocial care (45.1% of those who indicated past, present, or future use), compared to those with prostate cancer (22.5%), and other types (37.6%). It was also more common for users to be from another ethnicity (45.2%), rather than Canadian/European/British (35.6%). Interestingly, the rate of endorsement of using psychosocial resources was higher in those attending for their first visit (45.0%), than for those in active treatment (35.0%), or follow-up (33.6%). This may be due to the *intention* of the newer patients to use the services in the future, which may not translate into actual use. It may also mean that the screening programme gave them information about psychosocial services they otherwise would not have had until later in their treatment.

Finally, the relationship of distress caseness to psychosocial use was investigated. Of interest is that almost half of all patients identified as suffering significant distress levels on the BSI-18 have not used psychosocial resources in the past or present, and do not intend to use the services in the future. This percentage is highest for cases of somatisation (50.5% of cases not using psychosocial), followed by depression (42.5%) and anxiety (41.7%).

Other sources of support were also investigated, with high levels of endorsement of receiving support from family (87.5%) and friends (73.7%). These were followed by neighbours (24.2%), religious community members (22.3%), and other cancer centre staff (16.5%). Some patients also endorsed getting no support (4.5%). The relationship between distress and these other types of support was investigated by looking at the per cent of patients who identified as distress cases on the GSI within each group. Interestingly, there were no differences in the percentage of cases and noncases who indicated receiving support from family, friends, or community members. However, those who sought support from the Department of Psychosocial Resources (*χ*^2^=35.13, *P*<0.001), or an outside counsellor (*χ*^2^=6.35, *P*<0.05), were more likely to be identified as distress cases than those who did not seek such support. Specifically, 57.6% of those who visited psychosocial resources were identified as distress cases, and 49.5% of those who saw an outside counsellor. This is compared to an even distribution of about 37–40% of those both receiving and not receiving support from family, friends, and community members being identified as cases. This would seem to indicate that a greater proportion of those experiencing the most distress seek out professional, rather than community support. Nonetheless, even within this group, some 50% of the distress cases do not seek professional support.

## DISCUSSION

This study confirmed that a significant proportion of a large representative sample of diagnostically heterogeneous cancer patients self-reported clinical levels of distress, including anxiety and depression, across the disease continuum. Patients in this sample also reported high levels of fatigue and pain. A critical finding is that over one-third of all patients, whether currently in treatment or on long-term follow-up, continued to report significant levels of distress. This magnitude of distress is very similar to other reports, and also similar in terms of distress levels within different cancer diagnoses (Zabora *et al*., 2001a). The most distressed groups of patients included lung, pancreatic, Hodgkin's lymphoma, brain, head and neck, leukemia, and lymphoma patients.

Distress was also related to demographic characteristics including younger age, female gender, minority ethnicity, lower income, longer duration of illness, and being on active treatment or newly diagnosed. These latter two items may appear to be in opposition to one another: that is, having a new cancer diagnosis and/or being on active treatment would imply a shorter duration of illness, yet longer duration of illness was also independently predictive of higher distress. This may be consistent with a U-shaped distress curve over the course of the disease continuum, with higher levels of distress around the time of diagnosis, which abate somewhat following active treatment, but may rise as time goes by (Hanson *et al*., 2000). The ‘duration of illness’ variable may serve as a marker for disease recurrence or progression, as it is less likely that patients who were out of treatment, yet well, would be visiting the centre in great numbers. Unfortunately, accurate data on disease stage with which to verify this possibility is not available. This would be consistent with another study that found lower quality of life scores in palliative patients, compared to those in other stages of the disease continuum (Zabora *et al*., 1997). However, although quality of life was worse in these palliative patients, this study did not find significant differences in overall distress across the disease trajectory.

Previous reports indicate that younger patients may suffer higher levels of distress (Wenzel *et al*., 1999; Carlson and Bultz, 2002), theorised to be due to a larger disruption of social and familial roles at earlier developmental stages, such as raising small children and establishing careers. It may also be perceived as less ‘fair’ for a younger person to be struck by cancer, thereby causing additional distress. As well, patients at earlier life stages may have more limited life experience and problem-solving skills related to such a traumatic event. Similarly, historically socially disadvantaged groups such as women, minorities, and lower income earners also report higher distress levels as in this sample, perhaps illustrating that dealing with cancer involves more challenges for these groups. However, it is also possible that these disadvantaged groups would report higher distress levels in the general public as well, suggesting that a cancer diagnosis may add to an already heightened level of distress.

The high level of fatigue in this sample was also noteworthy, with almost half of all patients reporting fatigue as a problem. This is also consistent with other studies, and fatigue is beginning to be recognised as one of the most significant and long-term consequences of cancer and its treatment (Hann *et al*., 1997; Hann *et al*., 1999; Knobel *et al*., 2000; Okuyama *et al*., 2001). Of note is that almost half of all patients who were identified as suffering significant distress had not used psychosocial resources in the past or present, and did not intend to use the services in the future. This is of concern given that many empirically supported treatments for distress in cancer patients are widely available (for reviews, see Fawzy *et al*., 1995; Meyer and Mark, 1995; Bottomley, 1997; Iacovino and Reesor, 1997; Fawzy, 1999; Carlson and Bultz, 2003a). However, even though few sought formal professional care, most patients did endorse seeking support from family and friends. It appeared that those who were most in distress were more likely to seek professional, rather than community, support, which suggests that some people who most needed care were getting appropriate services.

There are certain methodological advantages that make this study unique. First, we managed to capture over 80% of eligible patients who passed through the centre over the designated time period (and perhaps more), which bolsters the generalisability of these results. The sample included a heterogeneous group of patients both in terms of type of cancer, and in terms of time since diagnosis and treatment phase, thus allowing direct comparisons between diagnoses and treatment phases. One of the major limitations of this study is its cross-sectional nature. It is not possible to draw any causal conclusions from the data, or to get a sense of how the same patients would fare prospectively as they moved from newly diagnosed onwards. Also, although relevant to a Canadian population, the generalisability of this data to other countries is not known. However, the similarities between this data and other reports from the USA and Europe help to bolster confidence in its universality.

The data also suggest that more needs to be done to change the face of clinical oncology practice when it comes to treating issues around emotional distress. It has been suggested that distress should be considered the ‘6th Vital Sign’, after blood pressure, temperature, respiration, pulse, and pain. This push could lead to more routine consideration of distress, in the same manner the pain community has raised awareness by referring to it as the ‘5th vital sign’. The NCCN has worked to develop distress screening and treatment guidelines, as outlined in the introduction, but the challenge now is to facilitate the implementation of screening and triage into routine clinical care. Some models have been successfully utilised in research settings including the use of computerised questionnaires, often web-based or using hand-held PDAs. The data supporting the feasibility of computer-based technology is convincing, in terms of its reliability, acceptability to patients, and utility in improving clinical care around psychosocial issues (Taenzer *et al*., 1997; Velikova *et al*., 1999; Taenzer *et al*., 2000; McLachlan *et al*., 2001; Carlson *et al*., 2001; Bezjak *et al*., 2001). This may be one avenue well worth pursuing in the effort to ensure that all patients are screened and appropriately referred for treatment of the distress that so commonly accompanies cancer diagnosis and treatment.

Treatment models themselves may also have to change in face of the patient profile that may result from the implementation of routine screening. With more broad-based screening efforts and less self-referral, the demographic may shift from primarily articulate, socially competent female patients towards those who are less verbal with fewer social skills, addiction histories, or compliance problems, who may not have sought out psychosocial support otherwise. The caseload may also shift from primarily women towards a more even balance of men and women, and more head and neck cancer patients. This patient population may not respond as well to the traditional ‘talk therapy’ often used to treat cancer-related distress, and be more amenable to behavioural and problem-solving interventions. Thus, the model of changing care may well look like the stages model suggested by Cunningham, who has identified a hierarchy of different types of group therapy, based on increasingly active participation by the recipient. The five types are described as: providing information, emotional support, behavioural training in coping skills, psychotherapy, and finally spiritual/existential therapy (Cunningham, 1995). Thus, a patient could begin by attending a large-group format psychoeducational workshop, and seek further intensive therapy if necessary, at their discretion.

This study serves to highlight a problem that has by now been well documented, and begs further directions and innovations in distress treatment. The best solution may be a marriage between appropriate screening using versatile technology, and programmatic assessment and triage at critical times during the cancer experience. If this can be integrated with a range of empirically based psychosocial treatment options, the most efficient and efficacious model of patient care is likely to result.

## References

[bib1] Bezjak A, Ng P, Skeel R, Depetrillo AD, Comis R, Taylor KM (2001) Oncologists' use of quality of life information: results of a survey of Eastern Cooperative Oncology Group physicians. Qual Life Res 10: 1–13 11508471 10.1023/a:1016692804023

[bib2] Blake-Mortimer J, Gore-Felton C, Kimerling R, Turner-Cobb JM, Spiegel D (1999) Improving the quality and quantity of life among patients with cancer: a review of the effectiveness of group psychotherapy. Eur J Cancer 35: 1581–1586 10673965 10.1016/s0959-8049(99)00194-x

[bib3] Bottomley A (1997) Where are we now? Evaluating two decades of group interventions with adult cancer patients. J Psychiatr Mental Health Nurs 4: 251–265 10.1046/j.1365-2850.1997.00060.x 9362828

[bib4] Canadian Association of Psychosocial Oncology (1999) Standards: Canadian association of psychosocial oncology. Canadian Association of Psychosocial Oncology, Montreal, Canada

[bib5] Carlson LE, Bultz BD (2002) Efficacy *vs*. cost of psychosocial interventions: an evidence-based call for action. Oncol Exchange 1: 34–51

[bib6] Carlson LE, Bultz BD (2003a) Benefits of psychosocial oncology care: improved quality of life and medical cost offset. Health Qual Life Outcomes 1: 8 12756059 10.1186/1477-7525-1-8 PMC155787

[bib7] Carlson LE, Bultz BD (2003b) Cancer distress screening: needs, methods and models. J Psychosomatic Res 55: 403–409 10.1016/s0022-3999(03)00514-2 14581094

[bib8] Carlson LE, Speca M, Hagen N, Taenzer P (2001) Computerized quality-of-life screening in a cancer pain clinic. J Palliat Care 17: 46–52 11324185

[bib9] Cunningham AJ (1995) Group psychological therapy for cancer patients. A brief discussion of indications for its use, and the range of interventions available. Support Care Cancer 3: 244–247 7551627

[bib10] Cunningham AJ (2000) Adjuvant psychological therapy for cancer patients: putting it on the same footing as adjunctive medical therapies. Psychooncology 9: 367–371 11038474 10.1002/1099-1611(200009/10)9:5<367::aid-pon473>3.0.co;2-i

[bib11] Derogatis LR (1983) SCL-90-R: Administration, Scoring and Procedures Manual-II, 2nd edn. Baltimore, MD: Clinical Psychometric Research

[bib12] Derogatis LR (1993) Brief Symptom Inventory: Administration, scoring, and procedures manual. Minneapolis, MN: National Computer Systems, Inc

[bib13] Derogatis LR (2001) Brief Symptom Inventory 18: Administration, Scoring and Procedures Manual. Minneapolis, MN: NCS Pearson Inc

[bib14] Derogatis LR, Morrow GR, Fetting J (1983) The prevalence of psychiatric disorders among cancer patients. J Am Med Assoc 249: 751–757 10.1001/jama.249.6.751 6823028

[bib15] Fawzy FI (1995) A short-term psychoeducational intervention for patients newly diagnosed with cancer. Support Care Cancer 3: 235–238 7551625 10.1007/BF00335895

[bib16] Fawzy FI (1999) Psychosocial interventions for patients with cancer: what works and what doesn't. Eur J Cancer 35: 1559–1564 10673962 10.1016/s0959-8049(99)00191-4

[bib17] Fawzy FI, Fawzy NW, Arndt LA, Pasnau RO (1995) Critical review of psychosocial interventions in cancer care. Arch Gen Psychiatry 52: 100–113 7848046 10.1001/archpsyc.1995.03950140018003

[bib18] Fawzy FI, Fawzy NW, Canada AL (1998) Psychosocial treatment of cancer: an update. Curr Opin Psychiatry 11: 601–605

[bib19] Fobair P (1997) Cancer support groups and group therapies: part I. Historical and theoretical background and research on effectiveness. J Psychosoc Oncol 15: 63–81

[bib20] Hann DM, Garovoy N, Finkelstein B, Jacobsen PB, Azzarello LM, Fields KK (1999) Fatigue and quality of life in breast cancer patients undergoing autologous stem cell transplantation: a longitudinal comparative study. J Pain Symptom Manag 17: 311–319 10.1016/s0885-3924(99)00007-x 10355210

[bib21] Hann DM, Jacobsen PB, Martin SC, Kronish LE, Azzarello LM, Fields KK (1997) Fatigue in women treated with bone marrow transplantation for breast cancer: a comparison with women with no history of cancer. Support Care Cancer 5: 44–52 9010989 10.1007/BF01681961

[bib22] Hanson FM, Suman VJ, Rummans TA, Dose AM, Taylor M, Novotny P, Johnson R, Evans RE (2000) Physical, psychological and social well-being of women with breast cancer: the influence of disease phase. Psychooncology 9: 221–231 10871718 10.1002/1099-1611(200005/06)9:3<221::aid-pon456>3.0.co;2-t

[bib23] Iacovino V, Reesor K (1997) Literature on interventions to address cancer patients' psychosocial needs: What does it tell us? J Psychosoc Oncol 15: 47–71

[bib24] Knobel H, Loge JH, Nordoy T, Kolstad AL, Espevik T, Kvaloy S, Kaasa S (2000) High level of fatigue in lymphoma patients treated with high dose therapy. J Pain Symptom Manag 19: 446–456 10.1016/s0885-3924(00)00144-5 10908825

[bib25] McLachlan SA, Allenby A, Matthews J, Wirth A, Kissane D, Bishop M, Beresford J, Zalcberg J (2001) Randomized trial of coordinated psychosocial interventions based on patient self-assessments *versus* standard care to improve the psychosocial functioning of patients with cancer. J Clin Oncol 19: 4117–4125 11689579 10.1200/JCO.2001.19.21.4117

[bib26] Meyer TJ, Mark MM (1995) Effects of psychosocial interventions with adult cancer patients: a meta-analysis of randomized experiments. Health Psychol 14: 101–108 7789344 10.1037//0278-6133.14.2.101

[bib27] National Comprehensive Cancer Network, Inc (2002) Practice Guidelines in Oncology. – v.1.2002: Distress Management. Version 1. 2002. Jentintown, PA: National Comprehensive Cancer Network, Inc 10.6004/jnccn.2019.0048 PMC6907687 31590149

[bib28] Okuyama T, Tanaka K, Akechi T, Kugaya A, Okamura H, Nishiwaki Y, Hosaka T, Uchitomi Y (2001) Fatigue in ambulatory patients with advanced lung cancer: prevalence, correlated factors, and screening. J Pain Symptom Manag 22: 554–564 10.1016/s0885-3924(01)00305-0 11516597

[bib29] Schneiderman N, Antoni MH, Saab PG, Ironson G (2001) Health psychology: psychosocial and biobehavioral aspects of chronic disease management. Annu Rev Psychol 52: 555–580 11148317 10.1146/annurev.psych.52.1.555

[bib30] Sellick SM, Crooks DL (1999) Depression and cancer: an appraisal of the literature for prevalence, detection, and practice guideline development for psychological interventions. Psychooncology 8: 315–333 10474850 10.1002/(SICI)1099-1611(199907/08)8:4<315::AID-PON391>3.0.CO;2-G

[bib31] Stefanek M, Derogatis L, Shaw A (1987) Psychological distress among oncology patients. Psychosomatics 28: 530–538 3432516 10.1016/S0033-3182(87)72467-0

[bib32] Taenzer P, Bultz BD, Carlson LE, Speca M, DeGagne T, Olson K, Doll R, Rosberger Z (2000) Impact of computerized quality of life screening on physician behaviour and patient satisfaction in lung cancer outpatients. Psychooncology 9: 203–213 10871716 10.1002/1099-1611(200005/06)9:3<203::aid-pon453>3.0.co;2-y

[bib33] Taenzer PA, Speca M, Atkinson MJ, Bultz BD, Page S, Harasym P, Davis JL (1997) Computerized quality of life screening in an oncology clinic. Cancer Pract 5: 168–175 9171553

[bib34] Velikova G, Wright EP, Smith AB, Cull A, Gould A, Forman D, Perren T, Stead M, Brown J, Selby PJ (1999) Automated collection of quality-of-life data: a comparison of paper and computer touch-screen questionnaires. J Clin Oncol 17: 998–1007 10071295 10.1200/JCO.1999.17.3.998

[bib35] Wenzel LB, Fairclough DL, Brady MJ, Cella D, Garrett KM, Kluhsman BC, Crane LA, Marcus AC (1999) Age-related differences in the quality of life of breast carcinoma patients after treatment. Cancer 86: 1768–1774 10547550

[bib36] Zabora J, BrintzenhofeSzoc K, Curbow B, Hooker C, Piantadosi S (2001a) The prevalence of psychological distress by cancer site. Psychooncology 10: 19–28 11180574 10.1002/1099-1611(200101/02)10:1<19::aid-pon501>3.0.co;2-6

[bib37] Zabora J, BrintzenhofeSzoc K, Jacobsen P, Curbow B, Piantadosi S, Hooker C, Owens A, Derogatis L (2001b) A new psychosocial screening instrument for use with cancer patients. Psychosomatics 42: 241–246 11351113 10.1176/appi.psy.42.3.241

[bib38] Zabora JR, Blanchard CG, Smith ED, Roberts CS, Glajchen M, Sharp JW, BrintzenhofeSzoc KM, Locher JW, Carr EW, Best-Castner S, Smith PM, Dozier-Hall D, Polinsky ML, Hedlund SC (1997) Prevalence of psychological distress among cancer patients across the disease continuum. J Psychosoc Oncol 15: 73–87

[bib39] Zabora JR, Smith-Wilson R, Fetting JH, Enterline JP (1990) An efficient method for psychosocial screening of cancer patients. Psychosomatics 31: 192–196 2330401 10.1016/S0033-3182(90)72194-9

